# Parental Stress and Disability in Offspring: A Snapshot during the COVID-19 Pandemic

**DOI:** 10.3390/brainsci11081040

**Published:** 2021-08-05

**Authors:** Martina Siracusano, Assia Riccioni, Leonardo Emberti Gialloreti, Eugenia Segatori, Lucrezia Arturi, Michelangelo Vasta, Maria Cristina Porfirio, Monica Terribili, Cinzia Galasso, Luigi Mazzone

**Affiliations:** 1 Department of Biomedicine and Prevention, University of Rome Tor Vergata, Via Montpellier 1, 00133 Rome, Italy; leonardo.emberti.gialloreti@uniroma2.it; 2 Child Neurology and Psychiatry Unit, Tor Vergata University Hospital, Fondazione PTV, Oxford Street 81, 00133 Rome, Italy; assiariccioni@gmail.com (A.R.); lucrezia.arturi@gmail.com (L.A.); michelangelovasta.roma@gmail.com (M.V.); mcporfy@gmail.com (M.C.P.); monica.terribili@gmail.com (M.T.); cinzia.galasso@uniroma2.it (C.G.); luigi.mazzone@uniroma2.it (L.M.); 3 Systems Medicine Department, University of Rome Tor Vergata, Montpellier Street 1, 00133 Rome, Italy; eugeniasegatori210@gmail.com

**Keywords:** ADHD, ASD, caregiver, COVID-19, disability, genetic syndrome, neurodevelopmental disorders, parents, Sotos, stress, Rett

## Abstract

Parenting a child with a disability, such as neurodevelopmental disorders and genetic syndromes, implies a high level of stress. During the COVID-19 outbreak—as a period implying additional challenges—few studies have specifically investigated caregivers’ distress among neurodevelopmental disabilities. The objective of the study is to investigate whether during the COVID-19 pandemic, the level of parental stress differs between four disability groups including neurodevelopmental disorders (autism spectrum disorder (ASD), attention deficit and hyperactivity disorder (ADHD)) and genetic syndromes (Rett syndrome (RTT), Sotos syndrome (SS)) in comparison to families with typical development offspring (TD). In total, 220 Italian parents of children affected by neurodevelopmental disabilities (74 ASD, 51 ADHD, 34 SS, 21 RTT, 40 TD; age M 9.4 ± SD 4.2) underwent a standardized evaluation for stress related to parenting through the self-report questionnaire, Parental Stress Index-Short Form (PSI-SF). The main findings show greater levels of parental stress—mainly linked to child behavioral characteristics rather than parental sense of competence—in parents of children affected by a disability in comparison to children with typical development. This study highlights the need to support not only individuals with special needs but also their own caregivers: core figures in the management and outcome of children disorders.

## 1. Introduction

Parents of children with disabilities, including neurodevelopmental disorders (i.e., autism, attention deficit hyperactivity disorder) and genetic syndromes, experience daily high levels of stress related to parenting.

The act of parenting a child with special needs implies several difficulties to be dealt with that may impact the caregivers’ quality of life [1,2,3]. Such difficulties include chronicity of child’s disability, therapeutical intervention cost and daily organization/schedule, management of daily school and after school activities, and lack of social support. Furthermore, specific clinical phenotypes related to child disability (i.e., motility and communication difficulties, maladaptive behaviors, sleep rhythms) and the level of impairment may be related to greater childcare challenges and higher parental stress [4,5].

Among neurodevelopmental disorders, autism spectrum disorder (ASD) and attention deficit hyperactivity disorder (ADHD) are characterized by developmental trajectory impairment (i.e., motor and language abilities, cognitive skills, behavioral profile) requiring a lifelong process of care (from first years of life up to adult age) with a resultant long-term impact on the parental well-being and quality of life of the whole family.

Parents of ASD children report greater rates of parental stress [6], and externalizing behaviors have been identified as the main clinical contributing factors to parenting related stress within families of young ASD children [4]. It is also well known that caregivers of children with ADHD experience a higher level of parental stress, and that the positive relationship between ADHD symptoms’ severity and parenting stress is clearly established [7,8].

In turn, the cognitive condition of a child, and in particular the presence or absence of intellectual disability (ID), may represent an additional burden for caregivers while dealing with a poorer adaptive children’s functioning and subsequently with higher dependence in relation to most of the daily life domains (i.e., use of environment, domestic behavior, health and safety, play, self-care, social abilities), experiencing higher parent-related stress [9].

In the context of neurodevelopmental disabilities, there are genetic syndromes—complex disorders caused by genetic mutations determining not only developmental trajectory impairment but also medical comorbidities (i.e., heart disease, gastrointestinal disorder, seizures)—that require additional medical follow-up and life-long pharmacological therapies. Within the wide spectrum of genetic syndromes, there are Rett syndrome and Sotos syndrome.

Rett syndrome (RTT) is an example of X-linked disorder (often caused by mutations in Methyl-CpG-binding protein 2 MECP2) always implicating severe intellectual disability with high level of dependency on parents due to profound motor, language and cognitive delay. In addition to this, RTT is associated with several medical problems (i.e., seizures, abnormal muscle tone, Scoliosis/kyphosis, breathing disturbances, sleep disorders, gastrointestinal difficulties) which require frequent hospitalization [10].

Sotos syndrome (SS) is a rare congenital overgrowth syndrome characterized by excessive height, weight and bone age and several organic defects (cardiac, genitourinary, scoliosis) [11]. Most SS individuals present a mutation of the gene encoding nuclear receptor-binding SET domain-containing protein 1 (NSD1) [12]. Neonatal hypotonia with consequent delayed developmental milestones (motor and language) is one of the earliest expressions of the disorder. Moreover, SS is associated with a heterogeneous neuropsychological phenotype—not yet well defined—with variable intellectual functioning and behavioral profile (impulsivity, atypical behavior) [13,14].

Therefore, within these genetic populations, caregivers may have to deal with additional childcare burdens such as recurring instrumental examination. In particular, regarding RTT, the main sources of parental stress were represented by medical concerns about their daughters’ health—seizures and gastrointestinal pain [15]. Furthermore, family’s psychological well-being of RTT individuals was negatively related to their level of behavioral problems [16] and girl’s age [17].

To the best of our knowledge, no previous study has specifically investigated stress related to parenting within Sotos syndrome.

During the COVID-19 pandemic, caregivers of children with neurodevelopmental disorders had to face not only the challenge of their children’s disability but also the pandemic.

Imposed home confinement, interruption of in-person interventions, school closure, remote working and job loss, represent extra difficulties for caregivers of children with disabilities belonging to several countries [18,19].

However, even if parents represent an essential figure in a child’s disability, few studies have specifically investigated—among neurodevelopmental disabilities—the relation between parenting and caregivers’ distress during the COVID-19 pandemic [20,21,22]. In fact, most of the research did not include clinical populations but only parents of children with typical development, not characterized by the additional challenges related to disability [23,24,25].

Adams et al. examined through an online survey, the changes in parental stress before (retrospectively evaluated) and after the pandemic outbreak (at two time points: May and September 2020) among 433 US parents of children aged 5–18 years [24]. The main finding of the study was an increase in parental stress (harder parenting if compared to pre-pandemic due to changes in children’s daily routine) over the initial course of the COVID-19 outbreak for 70% of participants and persisting up to longer distance (September 2020). However, given that no clinical information on offspring was provided, it is not clear if children were characterized by a typical development or if they presented any developmental, behavioral, or learning problems possibly influencing in any way the parental stress.

Interestingly, in a recent study on 1451 Chinese parents of children with special needs (including ASD, intellectual disability, hearing and visual impairment), parental stress was found as an effective predictor of parent’s anxiety status during the COVID-19 outbreak [21].

However, to measure parental stress, most studies employed online surveys [20,21] instead of a standardized measure such as the Parental Stress Index tool [26]. Moreover, to our knowledge, no previous research measured the stress related to parenting during the COVID-19 pandemic amongst Italian families with neurodevelopmental disorders and genetic syndromes.

## 2. Aim

We aimed to evaluate the level of parental stress during the COVID-19 pandemic through a standardized instrument—the Parental Stress Index-Short Form (PSI-SF)—applied to families with children and young adolescents affected by neurodevelopmental disorders and by genetic syndromes implying developmental disorders.

Specifically, we investigated whether the level of parental stress differed between four disability groups including neurodevelopmental disorders (autism spectrum disorder (ASD), attention deficit and hyperactivity disorder (ADHD)) and genetic syndromes (Rett syndrome (RTT), Sotos syndrome (SS)) in comparison to families with typical development offspring.

Finally, we evaluated if the presence of intellectual disability influenced the parental stress within children affected by neurodevelopmental disabilities. Specifically, we investigated whether the parental stress level differed among caregivers of children who presented cognitive impairment in comparison to children with an adequate intellectual functioning.

## 3. Materials and Methods

The study protocol was approved and registered (R.S. #216.20) by the local Institutional Review Board (Rome Tor Vergata University-Hospital Ethical Review Committee). Informed consent of a parent was obtained for each participant.

**Figure 1:**
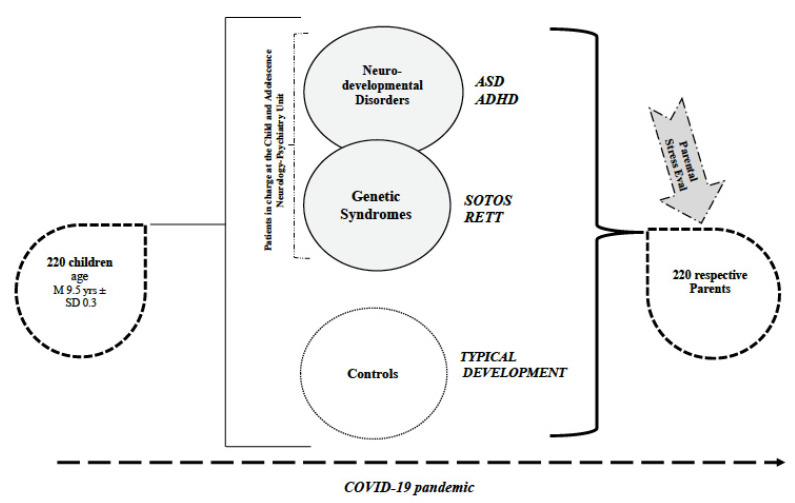
Overview of the Sample. Represented in the figure is the entire sample of the study as comprised of the study groups—individuals with neurodevelopmental disorders and genetic syndromes in charge at the Child and Adolescence Neurology Psychiatric Unit—and the typical development group. Parents of children belonging to each group underwent a Parental Stress Evaluation during COVID-19 pandemic. Legend: ASD = Autism Spectrum Disorder; ADHD = Attention Deficit and Hyperactivity Disorder; RETT = Rett Syndrome; Parental Stress Eval = Parental Stress Evaluation through Parental Stress Index-Short Form (PSI-SF).

The whole sample of the study included 220 participants, categorized in 4 study groups and 1 group of children with typical development (Figure 1).

Recruitment was performed during May–September 2020 (re-opening phase after the compulsory lockdown due to the COVID-19 emergency).

### 3.1. Participants

#### 3.1.1. Study Groups

To be eligible for the study, participants were required to have an age ranging from 2–19 years and:

- in regard to neurodevelopmental disorders: a diagnosis of ADHD or ASD, according to the Diagnostic and Statistical Manual of Mental Disorders-Fifth Edition criteria (DSM-5) [27].
- in regard to genetic syndromes: a diagnosis of Sotos syndrome (a confirmed diagnosis of SS by a clinical geneticist was available for all participants; but not all of them had a NSD1 gene mutation) or Rett syndrome (girls with typical RTT carrying MECP2 mutation and with clinical diagnosis according to Revised Diagnostic Criteria of RTT) [10].

All study group participants were recruited from the Child Psychiatry Unit of the University of Rome Tor Vergata Hospital, where they are in charge for developmental and behavioral problems and receive regular clinical follow-up, at least annually.

Members of our multidisciplinary team (child psychiatrists and psychologists) assessed for eligibility the individuals coming from the clinical database of our Unit, contacted the families by phone, described the study, and invited them to participate, planning a telehealth appointment (performed between October 2020 and December 2020).

#### 3.1.2. Typical Development Group

Children with typical development were voluntarily recruited during after-school activities prior to written informed consent of a parent or a legal guardian.

To be included as control, participants were evaluated by a child psychiatrist through clinical observation and the administration of the parental measure Child Behavior Checklist (CBCL) [28].

#### 3.1.3. Clinical Summary

The final sample of the study had a total of 220 children and young adolescents (67.3% male; 32.7% female; age M 9.4 ± SD 4.2; min 2, maximum 19 years) and the corresponding 220 parents (mean age M 45.2 ± SD 4.6; noteworthy, results were obtained mainly by mothers).

The whole sample consisted of four study groups, depending on the child’s diagnosis including, autism spectrum disorder (ASD), attention deficit and hyperactivity disorder (ADHD), Sotos syndrome (SS), Rett syndrome (RTT), and one group of typical development children (TD) (Table 1).

### 3.2. Procedure

A telehealth visit with a member of our multidisciplinary team was planned for parents belonging to the study groups (ASD, ADHD, RS, SS) and to the typical development group (TD), and was performed during October–December 2020.

During the telehealth appointment, parents of participants were asked to rate their level of stress through a specific self-report instrument: the Parental Stress Index (PSI-SF) [26].

### 3.3. Parental Stress Measure

The Parental Stress Index Short Form (PSI-SF) [26] is a self-report questionnaire developed from the long 120 items form, which measures the stress level of parents in their role as caregivers.

The PSI-SF includes 36 items grouped into 3 subscales (12 items each): parental distress (PD), dysfunctional parental–child interaction (D-PCI), and difficult child (DC). Specifically,

PD (items 1–12) provides a measure of stress related to parent characteristics, including feeling of competence, conflict with a partner, social support, restriction, and depression due to parenting.

D-PCI (items 13–24) evaluates parental satisfaction with the child and their relationship with him. A high score in this domain means that the parent perceives the child as unresponsive to his expectations, and that interactions with the child do not reinforce him as a parent.

DC (items 25–36) measures the parental difficulty in taking care of the child mainly due to child’s behavioral characteristics. Therefore, it is expected that parents of children with neurodevelopmental disorders report more stress in this domain.

Finally, the PSI-SF provides a PSI total subscale—sum of all scores—giving a measure of the overall stress of a person as a parent.

Most of the items (33) are rated using 5-point Likert scale: from 1 (strongly disagree) to 5 (strongly agree). Instead, 3 items (22, 32, 33) do not provide a Likert-type response choice.

A percentile score was measured for each subscale. Scores were corrected for age participants, according to the manual.

Specifically, scores equal or above the 90th percentile amount to a clinically significant stress for all subscales, except for P-CDI where 85 is already considered a significant cut off.

On the basis of the percentiles, the whole sample included in the study (4 study groups, 1 control group) was dichotomized as clinically stressed (CS) (≥90° for PD, DC and total; ≥85° for P-CDI) and non-clinically stressed (NCS) (<90° for PD, DC and total; <85° for P-CDI) parents.

Furthermore, according to the cut-off percentile reported in the PSI-SF manual, 2 parental stress variables were created to define if parents’ distress was related to parental competence (*Stress related to parents’proficiency* PD ≥ 90° + DC < 75°) or it was mainly due to child condition and difficulties in management of child behavioral features (*Stress related to Child Condition* PD ≥ 90° + DC >75°).

### 3.4. Cognitive Assessment

Based on the Intelligence Quotient (IQ) value, the study groups’ participants were dichotomized as “intellectual disability” (ID = IQ ≤ 70) and “no intellectual disability” (no ID = IQ > 70) (Table 1).

IQ assessment was performed in the context of previous clinical follow-ups in our Child and Adolescents Psychiatric Unit, through cognitive measures—chosen on the basis of age, expressive language level and cooperation of each participant—including Leiter International Performance Scale-Revised [29], the Wechsler Preschool and Primary Scale of Intelligence (Third Edition) (WPPSI-III) [30] or the Wechsler Intelligence Scale for Children (Fourth Edition) (WISC-IV) [31]. The same standard scores (SS = 100) and standard deviations (SD = 15) are used by all these measures. RTT participants did not undergo a standardized evaluation of IQ.

### 3.5. Child Behavior Checklist

The Achenbach Child Behavior Checklist (CBCL) [28] questionnaire was administered to the parents of individuals with typical development to exclude the presence of significant behavioral problems (internalizing and externalizing symptoms).

According to the age of their children, parents were administered the “18 months–5 years” or the “6–18 years” form. Caregivers were asked to rate their child’s adverse behavior on a 3-point Likert scale (0 = not true, 1 = sometimes true, 2 = often true) depending on the frequency of the behavior, with higher scores showing more problematic behavior. According to the T-scores the behavior is considered as typical (*T* < 65), borderline (*T* = 65–69), and clinically significant (*T* ≥ 70).

## 4. Statistical Analysis

Comparisons between groups were performed, as appropriate, with the independent samples *t*-test or Pearson’s χ^2^ test. Comparisons between more than two groups were examined by means of the one-way analysis of variance (ANOVA) and followed by post-hoc Welch two-sample *t*-test and Tukey contrasts for multiple comparisons of means. General linear models (GLMs) with tests for between-subject effects were used to test possible interactions between explanatory variables (age, type of disorder, intellectual disability). Binary logistic and nominal multiple regression models were developed to estimate the odds of parental distress (dependent variable) in relation to and after adjusting for possible predictive variables, such as age, sex, or intellectual disability of the child. An alpha level of 0.05 was used for all statistical analyses. Results, if not otherwise specified, are given as mean ± SD. All statistical analyses were performed using SPSS v.26.0 (IBM Corp., Armonk, NY, USA).

## 5. Results

### 5.1. Differences in Parental Stress Subscales

Using a one-way ANOVA test, a statistically significant difference emerged between the 5 groups, in regards to the percentiles of PSI-SF subscales *Dysfunctional Parent-Child Interaction* (F = 7.15; *p* < 0.001), *Difficult Child* (F = 10.80; *p* < 0.001) and *Total Stress* (F = 6.28; *p* < 0.001), while the *Parental Distress* difference was not statistically significant (F = 2.28; *p* = 0.062) (Table 1).

The post-hoc analysis revealed that the statistically significant differences were due to lower percentiles of the TD group, while no statistically significant differences were observed among the 4 study groups.

No statistically significant sex difference emerged in any Parental Stress Index Subscale percentiles.

### 5.2. Stressed vs. Non-Stressed: A Comparison between Groups

The following results are reported considering the whole sample dichotomized in *Clinical Stress* and *Non-Clinical Stress*, according to the cut-off percentile described in Section 3.

#### 5.2.1. Study Groups vs. Typical Development

We found statistically significant differences between study group (including all the disorders: ASD, ADHD, SS, RTT) and typical development group in the following subscales: *Dysfunctional Parent–Child Interaction Subscale* (χ^2^ = 14.122; *p* < 0.001), *Total Stress* (χ^2^ = 8.938; *p* < 0.003); *Difficult Child* (χ^2^ = 18.175; *p* <0.001); instead, *Parental Distress* did not emerge as a statistically significant difference (Table 2, part A).

#### 5.2.2. Study Groups: A Comparison between Disorders

Analyzing only the study groups no statistically significant results were found in the comparisons between ASD, ADHD, RTT and SS, in all the PSI subscales, except for *Parental Distress,* where a significant difference emerged (*p* = 0.002) (Table 2, part B).

As shown in Table 2 (part B) parents of ASD children were characterized by a higher percentage of clinically stressed participants (41.7%) in comparison to the other disorders. Parents of children with ADHD presented the lowest percentage of CS parents (9.8%).

### 5.3. Stress Related to the Child Condition or to Parents Proficiency

We then investigated the differences between groups, specifically concerning parental stress due to child condition (*Stress related to Child Condition*) or related to parental proficiency (*Stress related to Parental Proficiency*).

For these analyses, the variable named *Stress related to Child Condition* and *Stress related to Parental Proficiency,* were included according to the following cut-off percentiles. Specifically, the absence of parental stress (*No Parental Distress*) was considered for a PD score < 90°; *Stress related to Child Condition* for PD ≥ 90° + DC > 75°; and *Stress related to Parental Proficiency* for PD ≥ 90° + DC < 75°.

A statistically significant result emerged when comparing parents of children affected by disorders with parents of typical development children (study groups vs. TD) (χ^2^ = 26.506; *p* = 0.001) (Table 3). Then, statistically significant findings were observed also in the comparison between disorders (χ^2^ = 18.772; *p =* 0.005), and ASD emerged as the disorder characterized by more stress related to child condition (38.9% of parents); whereas ADHD presented the lowest percentage of parents stressed in relation to child condition (7.8%) (Table 3).

### 5.4. Differences in Parental Stress Considering the Presence or Absence of Intellectual Disability

We investigated if the cognitive condition of a child (presence or absence of ID) influenced the parental stress. For this reason, the whole sample (TD and study groups) was dichotomized in the presence or absence of ID. A significant difference in percentiles emerged between “parents of children with ID” and “parents of children without ID” in all PSI subscales (PD: t = 2.141, *p* = 0.033; DC: t = 2.397, *p* = 0.018; P-CDI: t = 2.592, *p* = 0.01; total stress: t = 2.074, *p* = 0.039) showing that the presence of ID significantly impacted caregivers’ stress in comparison to parents of children without ID.

To check for possible interactions between predictive factors, we performed a general linear model. We did not observe an interaction between ID and age of children in any PSI-SF subscale. In addition, no statistically significant interaction was observed between ID and disorder. The same general linear model showed that ID, after adjusting for age and disorder, continued to be associated with higher *Dysfunctional Parent–Child Interaction* percentiles (F = 5.182; *p* = 0.024). However, ID did not yield statistically significant results in the other PSI-SF subscales.

On the other hand, after adjusting for ID and age, the type of disorder was statistically significant and associated with higher percentiles in the *Dysfunctional Parent–Child Interaction Subscale* (F =5.077 *p* = 0.001), *Difficult Child* (F = 6.576; *p* < 0.001) and *Total Stress* subscale (F = 3.700; *p* = 0.006), but not for *Parental Distress* (F = 0.781; *p* = 0.539)

In a binary logistic regression model with dependent variable *Difficult Child* (clinical stress yes/clinical stress no), after adjusting for ID and sex, having a child with a disorder increased the odds of clinical stress by 6.8 (95% CI: 2.3–19.9). As for clinical stress related to *Dysfunctional Parent–Child Interaction Subscale*, the presence of a child with a disorder increased the odds by 5.9 (95% CI: 2.1–16.6). No increased odds were found for the other subscales.

A nominal logistic regression model, after adjusting for age and sex, showed an increased risk of parental distress related to child condition (OR = 5.1; 95% CI: 1.5–17.5). Contrarywise, no statistically significant increased risk was observed in relation to parental distress and to parental proficiency.

## 6. Discussion

In this study we investigated whether the level of parental stress during the COVID-19 pandemic differed among groups of children with neurodevelopmental disorders (autism spectrum disorder, attention deficit and hyperactivity disorder) and genetic syndromes (Rett syndrome, Sotos syndrome) in comparison to children with typical development.

Firstly, and as expected, we found that parents of children affected by a neurodevelopmental disability (study groups) reported significantly higher stress related to parenting if compared to peers with typical development. However, when comparing the level of parental stress with disabilities only a few significant results emerged, thus suggesting that during the COVID-19 pandemic (October–December 2020) all the disorders included in the study equally impacted the stress of caregivers.

Interestingly, this is concordant with a recent meta-analysis suggesting (even though not referring to the pandemic period) that having a child with a clinical disorder—and not a specific disorder—is a determining factor for parental stress [7].

Secondly, we evaluated if the parental stress that emerged within study groups was ascribable to a specific stress domain: parents’ proficiency (perceived parental competence) or child’s condition (stress linked to child clinical disorder; distress related to management difficulties of child behavior).

Coherently with what we expected, among all disorders, stress related to child condition represented the stress domain characterized by a greater percentage of affected parents. Interestingly, ASD children of our sample presented more stressed parents, with stress mainly arising from child’s characteristics (child’s condition) rather than from parental competence.

This is in line with previous studies, reporting children’s problematic behavior as a predictor of parental stress within ASD children [4,6,32]. In particular, Olson et al. [4] recently found externalizing behaviors as the main factors associated with stress in parents of 42 young ASD children (age range 15–67 months) in comparison to 36 typical development peers. The authors employed the PSI self-report questionnaire, similar to the instrument used in our research, but in the long version (120 items; PSI-4) [33]; however, their study was not conducted during the COVID-19 outbreak, and whilst including ASD as the only developmental disorder, it did not provide a comparison among disabilities as we did. Moreover, in line with our findings, a study including parents of ASD children and adolescents, discussed the increased and additional burden they had to face during the unexpected lockdown in their role as caregiver [34].

Noteworthy in our research, parents of ADHD participants emerged as being the ones with lower percentage of clinical stress (specifically concerning the two stress domains: parents’ proficiency and child condition) also considered in the comparison with parents of typical development children (no clinical stress: TD 80% vs. ADHD 90.2%). This unexpected finding may suggest that, within ADHD, during the pandemic the parental stress was not strictly related to perceived parents’ competence or child’s condition but was mainly due to a dysfunctional parental–child interaction meant as parental perception of a child as being unresponsive to her/his expectations. We may speculate that this feature results from difficulties in caretaking a child with ADHD symptoms (hyperactivity, need to stay in continuous movement and to be involved in activities) during the COVID-19 pandemic (quarantine, reduced opportunity of leaving home, closure of sport centers, school activities in remote modality).

Regarding genetic syndromes included in our research—RTT and SS—no specific parental stress profile emerged. However, even if no significant results were found, parents of RTT (children with greater developmental impairment, major dependence on parents and subsequent greater parental burden) unexpectedly reported the lowest percentage of clinical stress during COVID-19, if compared with other disorders. We may hypothesize that families who are used to greater daily stress (as parents of RTT individuals are), did not perceive a greater distress related to parenting during the pandemic, in comparison to other disabilities. However, to state this, an evaluation of parental stress before the pandemic outbreak is necessary.

Our results are not easily comparable with the available literature, because few studies investigated parental stress during the COVID-19 pandemic, and fewer still have examined this topic among parents of children affected by neurodevelopmental disorders and genetic syndromes.

Among works specifically conducted during the COVID-19 outbreak, there is Wang et al.’s [19] cross-sectional study on 1764 Chinese parents of children with ASD and 4962 parents of typically developing (TD) children. Parents were asked during March–April 2020, to complete an online survey specifically investigating the impact of anxiety and depression due to the COVID-19 crisis. Greater level of psychological distress, anxiety and depression problems emerged within parents of children with autism. However, the authors have seen the main limit of their study in the difficulty of disentangling between the ASD effect and the contextual COVID-19 effect.

In line with our results, a study by Levante et al. [22], performed during the pandemic, found higher parental distress (measured by self-reported Depression, Anxiety and Stress Scale questionnaire, DASS-21) within parents of 53 ASD children in comparison to 67 typical development peers. However, in this research a comparison between neurodevelopmental disorders was also not conducted, with ASD being the only disability included.

Finally, regarding the third objective of our study (to evaluate if the child’s cognitive condition—presence or absence of ID—influenced the parental stress), we found that overall, ID significantly impacted parental stress. However, looking at specific subscales, at equal child age and disorder, the *Dysfunctional Parent–Child Interaction* emerged as the only one significantly impaired by the cognitive condition of the child. This means that having a child with ID does not reinforce the person in his/her role as parent (interaction with the child does not respond to her/his expectations). Our finding is in line with a recent research conducted during COVID-19 on 515 Spanish individuals with a family member affected by intellectual disability. The authors reported that dysfunctional interaction patterns are among the family related factors that predicted parental stress [35].

However, interestingly, we found that having a child with a disorder (ASD, ADHD, RTT or SS), regardless of his cognitive level, significantly increased the stress level in most of the parental stress domains of the PSI-SF. This means that within our sample, the presence of the disorder (neurodevelopmental or genetic) is acting as an influence of parental stress as a whole, more than the presence or absence of ID.

The main strengths of our study are represented by: the use of a standardized tool such as PSI-SF to measure the parental stress; the inclusion of different groups of disability; and the presence of a control group of typical development children.

However, the present study is characterized by several limits: the employment of a parent report measure which does not offer an objective evaluation; the inclusion of a convenience sample (children with disabilities clinically followed by our unit); the lack of a previous stress evaluation prior the COVID-19 outbreak. Noteworthy is the fact that, whilst lacking an evaluation preceding the virus pandemic, this research does not intend to evaluate the impact of COVID-19 on parents of children affected by a disability. It is therefore impossible to affirm that the measured level of parental stress was mainly attributable to the pandemic itself, since we have not taken into consideration possible confounding factors, such as economic, family health, and duration of therapies interruption.

Our findings of greater stress related to parenting among families of children with disabilities (stress mainly linked to child behavioral characteristics rather than parental sense of competence) highlight the need to support not only individuals with special needs but also their own caregivers—core figures in the management and outcome of children disorders.

Future longitudinal studies are necessary for the disentanglement of the effective role of the COVID-19 pandemic from other factors potentially involved in influencing parental stress.

## 7. Conclusions

Our study represents a snapshot of the parental stress level present within Italian parents of children affected by neurodevelopmental disabilities (ASD, ADHD, RTT and SS) during a challenging period such as the COVID-19 pandemic.

The main conclusion of the study consists of the fact that, parenting a child with a developmental disorder—whichever this may be among the ones we considered— during the COVID-19 outbreak, has led to significantly higher caregiver stress, which is specifically linked to the child’s clinical disorder and therefore viewed as distress related to management difficulties of child disability.

## Figures and Tables

**Table 1:** Demographic characteristics of the sample and main results.

	ASD	ADHD	Sotos	Rett	TD	*F*	*p*-Value
*N*	74	51	34	21	40		
SEX (m/f)	62/12	42/9	18/16	0/21	25/15		
AGE (M ± SD)	7.9 ± 3.6	11.6 ± 2.2	9.9 ± 4.6	13.0 ±4.8	7.1 ± 3.9		
IQ (ID/no ID)	24/50	8/43	5/12 *	21	40		
*PARENTS’ DISTRESS* (PD) M ± SD	68.63 ± 31.85	58.82 ± 24.91	71.06 ± 29.69	71.48 ± 25.21	57.03 ± 28.43	2.28	0.062
*DYSFUNCTIONAL PARENT-CHILD INTERACTION* (DPCI) M ± SD	74.26 ± 25.27	78.82 ± 23.55	72.09 ± 27.19	71.00 ± 24.99	51.43 ± 29.24	7.15	**<0.001**
*DIFFICULT CHILD* (DC) M ± SD	76.78 ± 28.09	82.84 ± 19.88	78.56 ± 27.64	76.43 ± 24.19	48.45 ± 32.80	10.80	**<0.001**
*TOTAL STRESS* M ± SD	71.38 ± 29.50	76.47 ± 22.03	74.44 ± 27.68	71.43 ± 23.35	50.15 ± 30.0	6.28	**<0.001**

Demographic characteristics of the sample and main results of the Parental Stress Index-Short Form (PSI-SF) are summarized in the table. Mean percentiles are reported for each subscale of the PSI-SF (PD, DPCI, DC). In regards to cognitive functioning (IQ =Intellectual Quotient), the number of individuals affected by Intellectual Disability (ID) and not affected is reported (No ID). Significant differences in PSI-SF subscales between groups are reported in bold. * missing data for 17 Sotos participants.

**Table 2:** Stressed vs. Non Stressed.

	Parental Distress		Difficult Child		Disfunctional Parental Child Interaction		Total Stress	
	*No Stress*	*Clinical* *Stress*	*No Stress*	*Clinical* *Stress*	*No Stress*	*Clinical* *Stress*	*No stress*	*Clinical* *Stress*
**(A) Study Groups vs. Typical Development**								
TD	80.0%	20.0%	82.5%	17.5%	80.0%	20.0%	85.0%	15.0%
STUDY GROUP	70.8%	29.2%	45.3%	54.7%	47.2%	52.8%	60.0%	40.0%
	χ^2^ = 1.390 *p* = 0.238		χ^2^ =18.175 *p* < 0.001 *		χ^2^ = 14.122 *p* < 0.001 *		χ^2^ = 8.938 *p* = 0.003 *	
**(B) Comparison between Study Groups**								
ASD	58.3 %	41.7 %	45.2%	54.8%	48.6%	51.4%	54.1%	45.9%
ADHD	90.2 %	9.8 %	43.1%	56.9%	39.2%	60.8%	64.7%	35.3%
SOTOS	67.6 %	32.4%	44.1%	55.9%	50.0%	50.0%	55.9%	44.1%
RETT	71.4 %	28.6 %	52.4%	47.6%	57.1%	42.9%	76.2%	23.8%
	χ^2^ =14.857 *p* = 0.002 *		χ^2^ = 0.541 *p* = 0.910		χ^2^ = 2.307 *p* = 0.511		χ^2^ = 4.095 *p* = 0.251	

Shown in the table the percentage of parents with significant percentiles on the PSI-SF subscales: Parental Distress (PD), Difficult Child (DC), D-PCI (Dysfunctional Parental Child Interaction) and Total Stress. Table 2A reports the comparison between Study groups (all disorders) and Typical Development (TD). Table 2B reports the comparison among Study groups (ASD, ADHD, RTT, SS). statistically significant results are marked with *.

**Table 3:** Parent stress: Parents’ Proficiency vs. Child Condition.

	Parent Stress Related to Parents Proficiency	Parent Stress Related to Child Condition	No Parental Distress	*Comparison* *Study Groups vs. TD*	*Comparison between Study Groups*
TD	10.0%	10.0%	80.0%	χ^2^ = 26.506 *p* = 0.001 *	χ^2^ = 18.772 *p* = 0.005 *
ASD	2.8 %	38.9%	58.3%		
ADHD	2.0%	7.8%	90.2%		
SOTOS	2.9%	29.4%	67.6%		
RETT	9.5%	19.0%	71.4%		

Shown in the table the percentage of parents with: Parent stress related to Parents’ Proficiency (PD ≥ 90° + DC < 75°); Parent stress related to Child Condition (PD ≥ 90° + DC > 75°); No Parental Distress (PD < 90°). Statistically significant results (emerged from the comparison: between cases and typical development; between case groups) are marked with *. Legend: TD = Typical Development; ASD = Autism Spectrum Disorder; ADHD = Attention Deficit and Hyperactivity Disorder.

## Data Availability

The data presented in this study are contained within the article.
